# Modified cone bioassay methods for evaluating chlorfenapyr indoor residual spray

**DOI:** 10.5281/zenodo.22045349

**Published:** 2026-08-21

**Authors:** Jordan Benson, Frank S. C. Tenywa, Saphina Ngonyani, Aidi G. Lugenge, Jilly J. Mseti, Elingarami Sauli, Luca Facchinelli, Sarah J. Moore, Olukayode G. Odufuwa

**Affiliations:** 1 Vector Control Product Testing Unit, Environmental Health and Ecological Science Thematic Group, Ifakara Health Institute, P.O. Box 74, Bagamoyo, Tanzania.; 2 School of Life Sciences and Bioengineering, The Nelson Mandela African Institution of Science and Technology (NM-AIST), P.O. Box 447, Arusha, Tanzania.; 3 Swiss Tropical and Public Health Institute, Kreuzstrasse 2, 4123 Allschwil, Switzerland.; 4 University of Basel, Petersplatz 1, 4001 Basel, Switzerland.; 5 Liverpool School of Tropical Medicine, Pembroke Place, Liverpool, L3 5QA, England, United Kingdom.

## Abstract

**Background:**

Chlorfenapyr-based indoor residual spraying (IRS) has been prequalified for malaria control. Residual efficacy (mosquito mortality) of IRS applied to houses is typically assessed using forced-contact cone bioassays. However, chlorfenapyr is a pro-insecticide that requires mosquito metabolic activity for conversion to its toxic metabolite, tralopyril. Cone bioassays consistently underestimate mortality compared to free-flying bioassays. This study evaluated whether increasing mosquito activity during cone bioassays could better capture chlorfenapyr-induced mortality on mud surface using scalable methods.

**Materials and Methods:**

The bioefficacy of chlorfenapyr (Sylando® 240 SC) sprayed on mud surfaces was assessed in two experiments using cone bioassays against sugar-fed, laboratory-reared pyrethroid-resistant *Anopheles arabiensis* and *An. funestus*. Experiment 1 evaluated six exposure scenarios varying cone contact time (30 vs. 120 min) and post-exposure activity using clean CDC bottles (none, 1-hr vs. 12-hrs). Experiment 2 assessed the interaction between exposure duration (30-min vs. 12-hrs) and the presence or absence of a host (rabbit). Delayed mortality was monitored up to 120 hrs. An indicative mortality threshold was estimated using a random-effect meta-analysis of three free-flying semi-field studies.

**Results:**

Mortality remained low across all treatments in experiment 1 (7.8–15.4% at 72 hrs; 14.1–23.1% at 120 hrs), with no consistent improvement from post-exposure activity. In Experiment 2, mortality increased with 12-hrs exposure, particularly in the presence of a host (23.5% at 72 hrs; OR 2.25, 95% CI 1.71–2.97, *p* < 0.001). However, mortality remained well below the indicative value (52%).

**Conclusions:**

Prolonged exposure and host presence modestly increased mortality, but did not produce values consistent with expected chlorfenapyr bioefficacy against free-flying mosquitoes. These findings suggest that modified cone bioassays are unlikely to provide reliable estimates of chlorfenapyr-IRS performance. Further validation is required to assess whether overnight cone tests in people's homes can provide a more realistic measure of residual bioefficacy.

## Introduction

Indoor residual spraying (IRS), which involves applying insecticide to indoor surfaces such as walls and ceilings where mosquitoes rest [1], has contributed significantly to malaria reduction over the past decades [2]. IRS played a major role in malaria eradication in several countries [3], including recent successes in China and Egypt [4,5]. Sustained IRS use, significantly reduces malaria morbidity [6,7], and is useful for insecticide resistance management when combined with insecticide-treated nets (ITNs) with a different mode of action [8]. Given persistent malaria transmission and increasing insecticide resistance [9], maintaining effective IRS options from multiple insecticide classes remains essential.

However, resistance to commonly used insecticide classes, including pyrethroids, carbamates, and organophosphates, has weakened malaria vector control efforts [10,11], highlighting the need for new insecticide classes [12]. Chlorfenapyr is a novel pro-insecticide effective against pyrethroid-resistant malaria vectors [13–15], which acts by disrupting mitochondrial respiration in mosquitoes, causing energy depletion, paralysis and death [16]. Initially developed for agricultural use, chlorfenapyr has demonstrated high efficacy (mosquito mortality) against pyrethroid-resistant mosquitoes when formulated for IRS [14,17–19]. In 2024, the World Health Organization (WHO) prequalified Sylando® 240 SC, a chlorfenapyr-based IRS product for public health use [20].

IRS products undergo thorough testing, and are often assessed using a standard 30-min cone bioassay. Residual bioefficacy is generally considered satisfactory when mosquito mortality reaches ≥ 80% within the product specific assessment period [1,21]. However, chlorfenapyr-based IRS formulations failed to meet this WHO threshold in WHO Pesticide Evaluation Scheme (WHOPES) reviews, with 24-hrs mosquito mortality ranging from 22% to 69% in the first four months post-spraying [22]. This is likely because chlorfenapyr is a pro-insecticide best evaluated in free-flying bioassays where mosquitoes are sufficiently metabolically active to convert chlorfenapyr into its active metabolite, tralopyril [18,23]. The tunnel test is considered particularly reliable for such insecticides used in ITNs [23], but it is impractical for operational monitoring of IRS.

This study aimed to support the development of a simple, low-cost and robust bioassay that could be implemented by National Malaria Control Programmes (NMCPs) to monitor the residual efficacy of chlorfenapyr-based IRS products under operational conditions. To achieve this, two experiments were conducted. First, the standard Centres for Disease Control and Prevention (CDC) bottle bioassay can reliably assess chlorfenapyr bioefficacy, ensuring uniform exposure, movement and allowing sufficient time for metabolic activation [24]. Building on this principle, the present study evaluated a modified “flight cone” bioassay incorporating a post-exposure activation phase. Mosquitoes exposed to chlorfenapyr-treated mud surfaces for 30 or 120 minutes were transferred either directly to holding cups or into clean CDC glass bottles for 1 or 12 hrs in an attempt to stimulate movement, and promote conversion of chlorfenapyr into its active toxic form before mortality assessment [24]. The second experiment assessed the effect of host cues during exposure using four treatment permutations: 30-min cone exposure with or without the presence of a rabbit, and 12-hrs cone exposure with or without a rabbit. Mosquito mortality was measured at 24-hr intervals up to 120 hrs after exposure. To ensure consistent application of Sylando® 240SC, a track sprayer was used [25].

## Materials and Methods

The study was conducted at the Vector Control Product Testing Unit (VCPTU) facility of the Ifakara Health Institute (IHI) in Bagamoyo (“6°8’ S, 30°37’ E”), Tanzania.

### Study design

An iterative study design was used to investigate the optimum cone bioassay method for evaluating the bioefficacy of chlorfenapyr-IRS in the laboratory. Two experiments were conducted with the aim of increasing mosquito activity post cone exposure in order to increase mosquito mortality: The first experiment assessed two cone exposure durations (30 or 120 min) on chlorfenapyr-treated surfaces, followed by either no additional post-exposure activity or post-exposure mosquito activity in a clean CDC glass bottle for 1 or 12 hrs (flight cone bioassay). The experiment had six exposure arms:

1. 30-min cone exposure
2. 120-min cone exposure
3. 30-min cone exposure + 1-hr CDC bottle confinement
4. 30-min cone exposure + 12-hrs CDC bottle confinement
5. 120-min cone exposure + 1-hr CDC bottle confinement
6. 120-min cone exposure + 12-hrs CDC bottle confinement

Based on the results of the first experiment, a second experiment was conducted to assess the effect of cone exposure duration (30-min or 12-hrs exposure) in the presence and absence of a rabbit host in a test room. The experiment had four exposure arms:

1. 30-min cone exposure without a rabbit
2. 30-min cone exposure with a rabbit
3. 12-hr cone exposure without a rabbit
4. 12-hr cone exposure with a rabbit

### Mosquitoes

Two pyrethroid-resistant species were used: *Anopheles arabiensis* (Kingani strain) and *An. funestus* (FUMOV strain), being the primary malaria vectors in Tanzania [26,27]. Mosquitoes were aged 3-5 days post-emergence and sugar-fed. Following WHO guidelines,the susceptibility profiles of the study mosquitoes were confirmed in February 2025 using WHO tube tests for pyrethroids and CDC bottle bioassays for chlorfenapyr (Table 1).

**Table 1:** Resistance profile of laboratory colonies measured by mortality.

Mosquito species	Strain	Year of colonisation	Test date (2025)	Testing modality			
				Insecticide dose	Deltamethrin (0.05%)	Alpha-cypermethrin (0.05%)	Chlorfenapyr 100mg/ml
				Method	WHO tube test	WHO tube test	CDC bottle bioassay
*An. arabiensis*	Kingani	2005	21/02	% Mortality*	70%	62%	96%
*An. funestus*	Fumov	2018	13/02	% Mortality*	52%	34%	100%

* Deltamethrin and alpha-cypermethrin mortality recorded after 24 hrs; chlorfenapyr after 72 hrs

All colonies were maintained at the Bagamoyo insectary by feeding larvae with Tetramin® fish flakes (Tetra GmbH, Melle, Germany) evenly dispersed on the water surface using a spoon and kept at a density of 200 larvae per L of distilled water. Adult mosquitoes were kept in 30x30x30 cm cages and were allowed to feed on 10% sucrose solution *ad libitum*. For egg production, adult mosquitoes were given cow blood through a membrane paper cup feeder. The insectary was maintained at a temperature of 27 ± 2°C and a relative humidity of 75 ± 10%, under a 12:12 ambient light-dark cycle in line with MR4 guidance [28].

### Insecticide and its application

Sylando® 240SC is a suspension concentrate containing 240 g/L chlorfenapyr (21.45%) as the active ingredient. It was applied using a calibrated horizontal track sprayer (manufactured by Micron Sprayers Ltd., Herefordshire, UK) on mud surfaces (being the most commonly used surface for housing in village settings [29]) at a target dosage of 250 milligrams (mg) active ingredient per square metre (A.I./m²). The product is manufactured by BASF Agricultural Solutions GmbH, Germany, to be used in IRS for the control against pyrethroid-resistant mosquitoes. Untreated control mud panels were sprayed with water using a micron track sprayer.

### Preparation of mud panels

**Figure 1:**
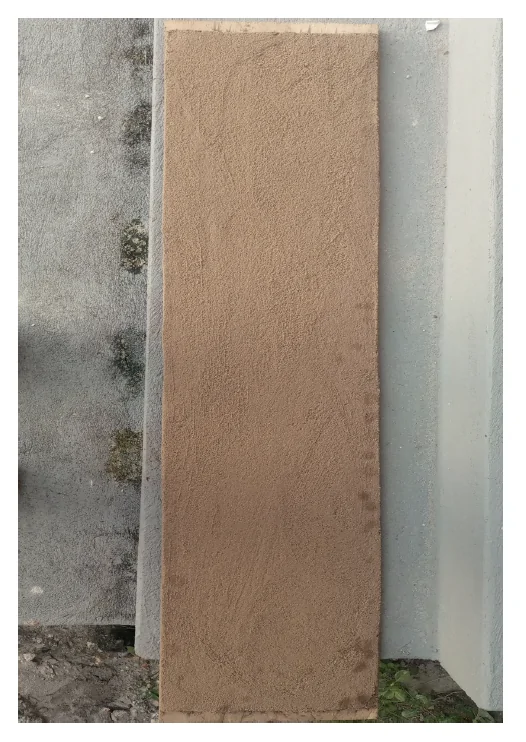
Mud panel preparation.

A total of 14 mud panels (Figure 1) were prepared by cutting wood frames to uniform dimensions of 119.2 cm in length and 24 cm in width, thereby minimising variation between batches. The soil and sand were sieved separately to remove stones, gravel, and debris. The soil was sourced from the nearby village of Mtoni, the sand from the location of the facility, and deionised water from the insectary. Each mud panel was prepared according to internal Standard Operating Procedure (SOP) for substrate preparation using a ratio of 2 parts soil, 3 parts sand and 1.25 parts deionised water, corresponding to 2473.2 g of soil, 3709.8 g of sand, and 1546.9 mL of deionised water per panel. The three constituents were mixed and stirred vigorously for 3-5 min until the mixture was uniform. The mixture was then used to fill each panel to a thickness of 1 cm. A trowel was used to level and smooth the surface. This process was repeated for all panels, which were stored in the untreated materials store at 25.8 °C (IQR: 25.4-26.4) and 89.6% (IQR: 87.2-92.6) relative humidity and allowed to dry for at least 14 days before spraying. Before spraying, the surface of mud panels was scraped to measure pH, which was confirmed to be near-neutral (pH 7.5). After spraying, the panels were stored in the treated materials store at 27.3 °C (IQR: 26.5-27.8) and 84.4% (IQR: 81.5-87.8) relative humidity until testing.

### Micron track sprayer calibration

**Figure 2:**
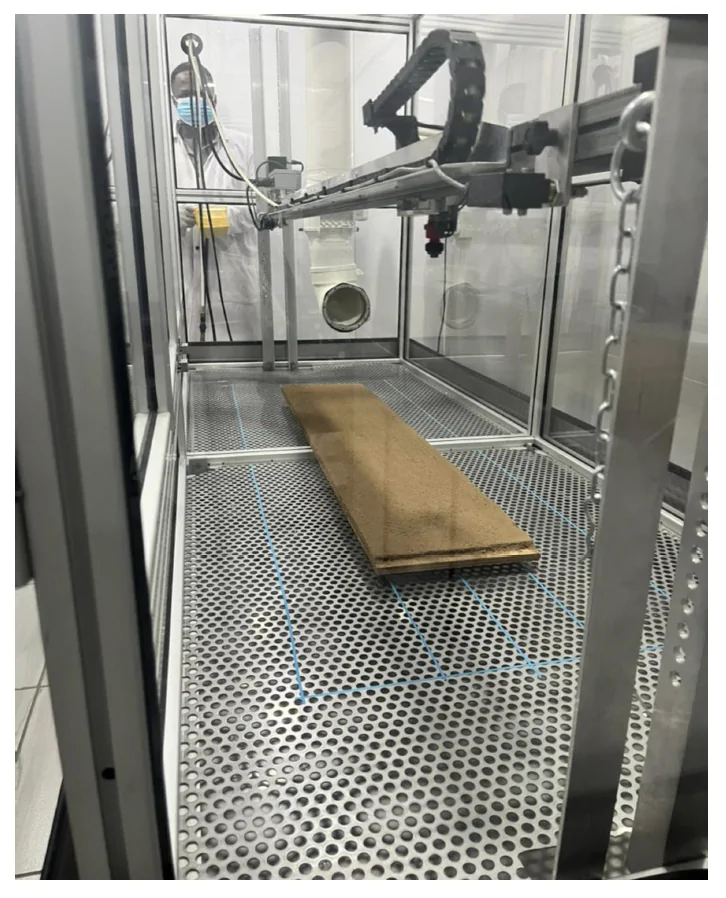
Spraying of the mud panel using the track sprayer.

The horizontal Micron track Sprayer [25] was calibrated to an application rate of 30 ml/m^2^. This calibration involved a two-step process: adjusting both the track speed and the nozzle flow rate. First, the sprayer was set up with a TeeJet DG95015EVS nozzle (90-degree flat fan, 40 psi pressure, 45 cm height). The track speed was then calibrated to 0.30 m/s by timing its 1.6-metre movement. Next, the flow rate was confirmed by collecting the spray for 30 sec, yielding approximately 200 mL/min (±10 mL/min). To ensure the final application was accurate, fluorometry was used. Filter papers were sprayed and the fluorescent dye deposited was measured against a calibrated curve. This confirmed that the deposition volume was within ±10% of the target, and track speed adjustments were made as needed to achieve the desired volume (Figure 2).

### Cone bioassay procedure

The standard cone bioassay was used as the reference comparator for the modified exposure and post-exposure holding conditions tested in this study [1]. For each cone bioassay replicate, 10 mosquitoes were exposed. Each species was exposed into separate cones using a siphon, and the cones were temporarily sealed with a plastic bung made from cotton wool inserted into the tip of a latex glove [21]. Mosquitoes were exposed to the insecticide and later transferred to a paper cup to assess mortality. All mosquitoes in the paper cups were provided with a 10% sugar solution and kept in a controlled environment (27±2^ ∘ ^C, 80±10% relative humidity) following WHO guidelines [21], to monitor mortality at 72 and 120 hrs. All tests were conducted in the evening from 17:00 hrs onwards to account for the mosquitoes' circadian rhythm [30]. Metabolic activity up-regulates at night [31], which influences both their detoxification and activation of chlorfenapyr [23].

### Experimental procedures

#### Experiment 1: Exploring the flight cone bioassay

**Figure 3:**
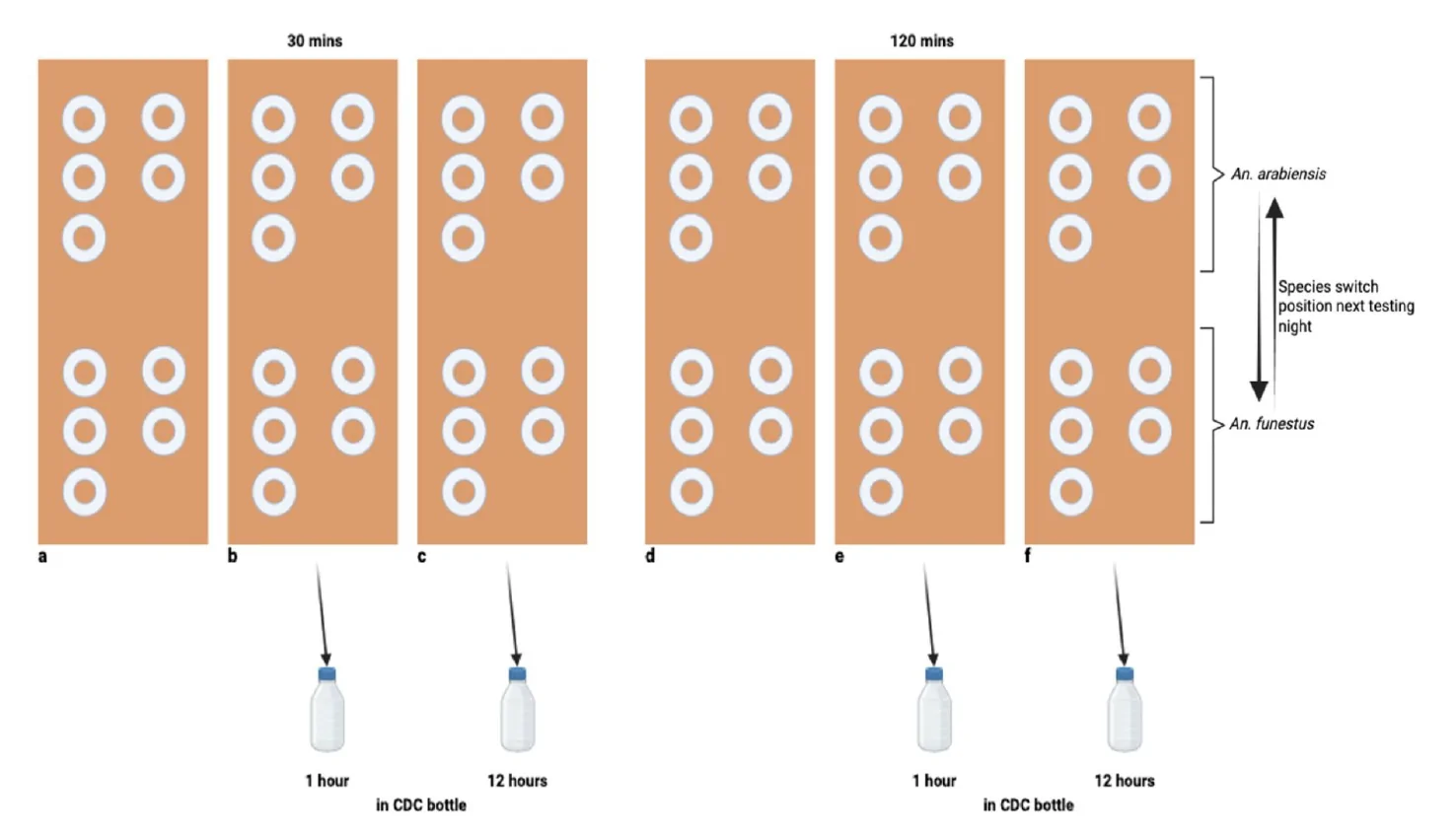
Flight cone bioassay schematic setup. Panels a–f represent treatment arms comprising: (a) 30-min cone exposure; (b) 30-min cone exposure + 1-hr CDC bottle confinement; (c) 30-min cone exposure + 12-hrs CDC bottle confinement; (d) 120-min cone exposure; (e) 120-min cone exposure + 1-hr CDC bottle confinement; and (f) 120-min cone exposure + 12-hrs CDC bottle confinement.

Experiments were conducted using 12 mud panels positioned at a 90° angle to mimic the vertical wall surfaces of rural houses under operational conditions (six mud panels were sprayed with Syllando® 240SC, and the other six mud panels were sprayed with water as controls). Mosquitoes were exposed to the insecticide for either 30 min (following the standard WHO protocol) or 120 min. To assess the performance of each of the treatment arms, the mosquitoes were processed in two different ways: 1) Mosquitoes were immediately aspirated into a paper holding cup after exposure; 2) Flight activity – mosquitoes were first moved to a clean CDC glass bottle [24] for either 1 or 12 hrs in an attempt to stimulate flight activity before they were transferred to a paper cup to assess mortality. The experiment was conducted over 8 nights from June 19th to July 14th, 2025. Each night, five cones per treatment arm were used, as well as a negative control for each treatment arm of the experiment (Figure 3). To ensure standardisation, experiments consistently started with the 120-min exposure arms, followed 1 hour later by the 30-min exposure arms. To control for positional bias on the mud panels, the allocation of species to the top or bottom locations was alternated each night. After a two-nights break, the sequence was repeated for nights 5-8.

#### Experiment 2: Influence of a host (rabbit) in the test room

**Figure 4:**
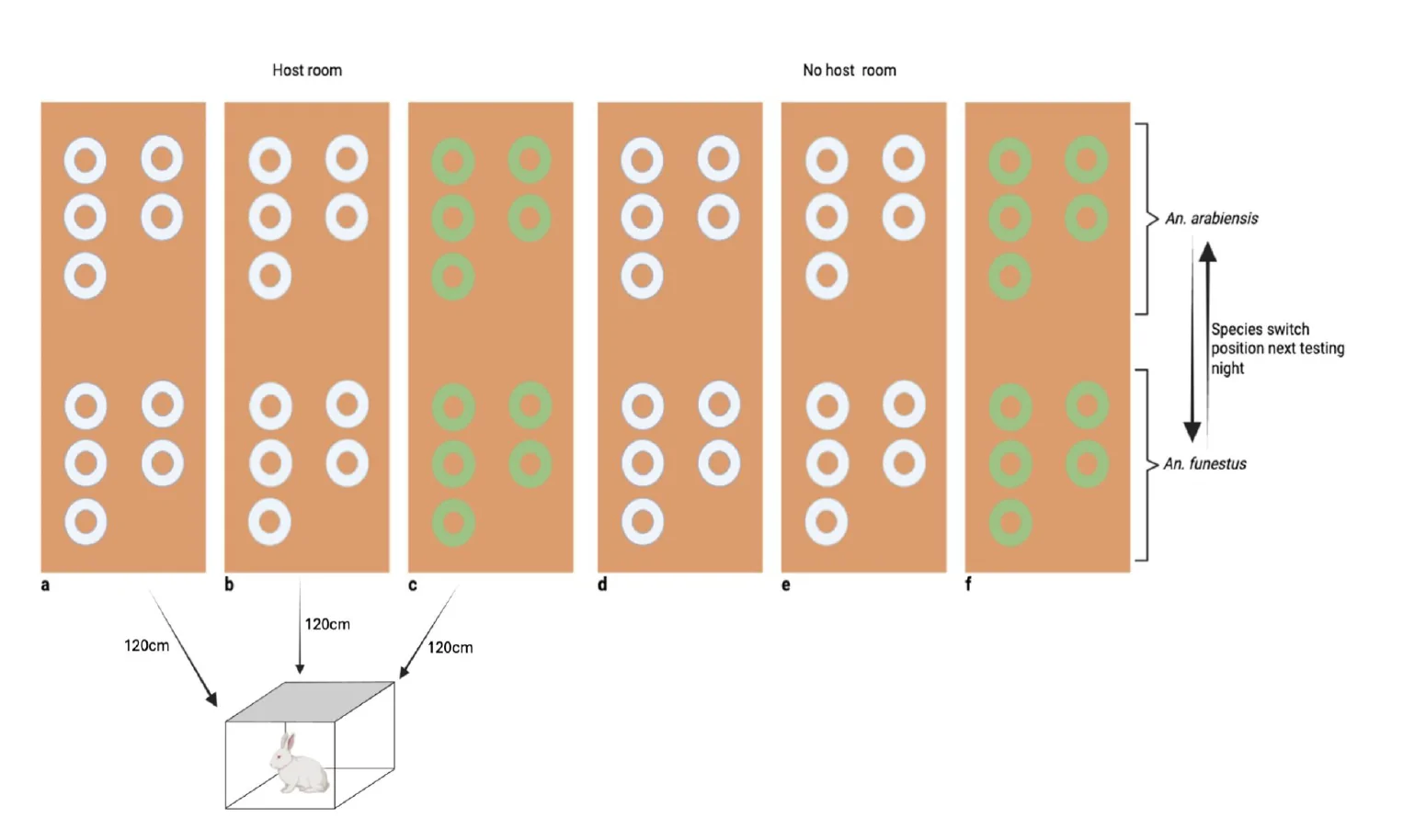
Schematic of the host-cone bioassay setup. Panels (a) and (b) represent treated mud surfaces and (c) the control within a room containing a host; while (d) and (e) represent treated mud surfaces and (f) the control in the room with no host.

It was assumed that the presence of a host might increase mosquito movement within the cones, enabling more conversion of the pro-insecticide to its metabolite. Therefore, to assess the performance of the cone-host bioassay in estimating the bioefficacy of chlorfenapyr-IRS in the presence of a rabbit host, the tests were conducted over 4 nights (24-28th November, 2025) using 2 test rooms. These rooms were separated by a central buffer room to ensure independence of observations (Figure 4). Each night, three mud panels were positioned at a 90° angle in each room. The experimental setup evaluated two distinct conditions:

Host Room: This room contained two chlorfenapyr-treated mud panels, one untreated mud panel, and a caged rabbit. The panels were positioned about 120 cm away from the rabbit.

No-Host Room: This room had the same mud panel setup (two treated, one untreated) but did not contain a rabbit.

In each test room, five cones per treatment arm were used. To account for any positional bias, the species’ locations on the mud panels (top or bottom) were alternated each night. Each night, mosquitoes were exposed to one of two exposure experimental arms for 30 min or 12 hrs. Following exposure, they were moved to paper cups with a 10% sugar solution, held under standard conditions with mosquito mortality assessed at 72 and 120 hrs.

### Data management and statistical analysis

Data were collected using paper forms and double-entered into Microsoft Excel. Data were cleaned and analysed using STATA statistical software, version 18.0 [32]. Data were cleaned by checking the balance of arms, replicates and number of mosquitoes exposed, alive and dead. Descriptive statistics were used to summarise the number of mosquitoes exposed, the number of mosquitoes dead and the percentage arithmetic mean mortality with corresponding 95% confidence intervals per treatment arm, with data were presented in tables and figures. The experiment was considered valid when mortality in the negative control did not exceed 20% at 72 hrs after exposure.

Binomial logistic regression was performed to assess mosquito mortality at 72 hrs (M72) as the primary outcome and 120 hrs (M120) as the secondary outcome to compare the exploratory exposure arms against the standard 30-min cone bioassay. The regression models were performed on pooled (all species) mortality, adjusted for mosquito species (*An. arabiensis* and *An. funestus*), and the day of the experiment as fixed effects, because both factors have been shown to be sources of variation in cone tests [33]. For the species-specific subgroup analyses (presented in Supplementary Tables 1 and 2), day of the experiment was a fixed effect.

**Table 2:** Delayed mortality at 72 and 120 hrs of *An. arabiensis* and *An. funestus* following cone exposure on chlorfenapyr-treated mud surfaces and CDC bottle confinement.

72 hrs holding time (Combined *An. arabiensis* and *An. funestus*)						
Treatment arms		Total exposed (400 per test day)	No Dead	% Arithmetic mean mortality (95% CI)	OR* (95% CI)	p-value
Cone-exposure time (mins)	Post-exposure activity time (hrs) in a clean CDC bottle					
30	None	800	94	11.8 (8.0 – 15.5)	1.00	
120	None	800	71	8.9 (6.1 – 11.7)	0.73 (0.52 – 1.01)	0.056
30	1 CDC	800	66	8.2 (5.3 – 11.2)	0.67 (0.48 – 0.93)	0.019
120	1 CDC	800	62	7.8 (5.1 – 10.4)	0.62 (0.44 – 0.88)	0.007
30	12 CDC	800	123	15.4 (12.0 – 18.8)	1.38 (1.03 – 1.85)	0.032
120	12 CDC	800	97	12.1 (8.9 – 15.3)	1.04 (0.76 – 1.41)	0.814
120 hrs holding time (Combined *An. arabiensis* and *An. funestus*)						
30	None	800	155	19.4 (14.6 – 24.2)	1.00	
120	None	800	123	15.4 (11.9 – 18.9)	0.75 (0.57 – 0.97)	0.031
30	1 CDC	800	137	17.1 (12.8 – 21.5)	0.85 (0.66 – 1.11)	0.233
120	1 CDC	800	113	14.1 (10.3 – 17.9)	0.67 (0.51 – 0.88)	0.004
30	12 CDC	800	185	23.1 (18.9 – 27.3)	1.27 (0.99 – 1.62)	0.060
120	12 CDC	800	176	22.0 (17.9 – 26.1)	1.18 (0.92 – 1.52)	0.183

* Adjusted Odds Ratio

Exploratory arms were assessed to identify mortality proportions within ±5% of the meta-analysis-derived indicative value for chlorfenapyr-IRS cone bioassays (Supplementary Table 3). This value was established because chlorfenapyr-IRS cone tests typically fall below the historical 80% mosquito mortality WHO threshold [14,15,18]. It was modelled using a random-effects framework that pooled 72-hr mosquito mortality proportions from three eligible studies: one miniature experimental hut (MEH) that is within the study site and two trials in experimental huts from Benin, adjusting for study-level variations as a random effect [14,18,19].

**Table 3:** Delayed mortality at 72 and 120 hrs of *An. arabiensis* and *An. funestus* following cone exposure on chlorfenapyr-treated mud surfaces with and without rabbit.

72 hrs holding time (Combined *An. arabiensis* and *An. funestus*)					
Treatment arms	Total exposed (400 per test day)	No Dead	% Arithmetic mean mortality (95% CI)	OR^a^ (95% CI)	p-value
30 min exposure; no rabbit	800	101	12.6 (9.1 – 16.1)	1.00	
30 min exposure; with rabbit	800	81	10.1 (7.6 – 12.6)	0.77 (0.56 – 1.06)	0.107
12 hrs exposure; no rabbit	800	123	15.4 (11.5 – 19.3)	1.28 (0.95 – 1.71)	0.103
12 hrs exposure; with rabbit	800	188	23.5 (18.7 – 28.3)	2.25 (1.71 – 2.97)	<0.001
120 hrs holding time (Combined *An. arabiensis* and *An. funestus*)					
30 min exposure; no rabbit	800	181	22.6 (17.5 – 27.8)	1.00	
30 min exposure; with rabbit	800	192	24.0 (19.7 – 28.3)	1.09 (0.85 – 1.39)	0.494
12 hrs exposure; no rabbit	800	219	27.4 (22.6 – 32.1)	1.33 (1.04 – 1.68)	0.021
12 hrs exposure; with rabbit	800	255	31.9 (26.7 – 37.1)	1.69 (1.34 – 2.14)	<0.001

* Adjusted Odds Ratio

## Results

In all experiments, the temperature and relative humidity within the testing and holding rooms were maintained between 27 ± 2°C and 80 ± 20%, respectively, in line with the WHO guidelines. Negative control mortality was 0.13% (95% CI: 0.08 – 0.17) at 72 hrs in flight cone bioassay, and 1.63% (95% CI: 1.29 – 1.96) at 72 hrs in the host experiment; therefore, mortality was not control-corrected [34].

### Delayed mosquito mortality following flight cone bioassay exposure

Overall, mortality responses were modest and inconsistent, with no clear improvement associated with increased exposure duration or short-term post-exposure activity, while longer post-exposure activity produced moderate increases in delayed mortality (Table 2).

### 72-hours mortality

At 72 hrs post-exposure, mortality was low across all treatment conditions, ranging from 7.8% to 15.4%. Compared with the reference condition (30-min cone exposure without post-exposure activity; 11.8% mortality), mortality was significantly reduced after both 30 and 120-min exposures followed by 1 hr in a clean CDC bottle (OR = 0.67, 95% CI: 0.48–0.93, *p* = 0.019; and OR = 0.62, 95% CI: 0.44–0.88, *p* = 0.007, respectively). A similar, borderline reduction was observed with 120-min exposure without post-exposure activity (8.9% mortality; OR = 0.73, 95% CI: 0.52–1.01, *p* = 0.056). In contrast, extending post-exposure activity to 12 hrs significantly increased mortality to 15.4% after 30-min exposure (OR = 1.38, 95% CI: 1.03–1.85, *p* = 0.032), but had no effect following 120-min exposure (12.1%; OR = 1.04, 95% CI: 0.76–1.41, *p* = 0.814) (Table 2).

### 120-hours mortality

At 120 hrs post-exposure, mortality was slightly increased across all treatments (14.1%–23.1%), (Table 2) and greater differences between treatment arms were observed. Relative to the reference condition (30-min exposure with no activity; 19.4% mortality), mortality was significantly lower after 120-min exposure without activity (15.4%; OR = 0.75, 95% CI: 0.57–0.97, *p* = 0.031) and 120-min exposure plus 1 hr of activity (14.1%; OR = 0.67, 95% CI: 0.51–0.88, *p* = 0.004). No significant difference was observed for 30-min exposure with 1 hr of activity (17.1%; OR = 0.85, 95% CI: 0.66–1.11, *p* = 0.233). In contrast, 12 hrs of activity slightly increased mortality following both 30 and 120-min exposures but this was not significant (23.1% and 22.0%; OR = 1.27, 95% CI: 0.99–1.62, p = 0.060; and OR = 1.18, 95% CI: 0.92–1.52, p = 0.183, respectively) (Table 2).

**Figure 5:**
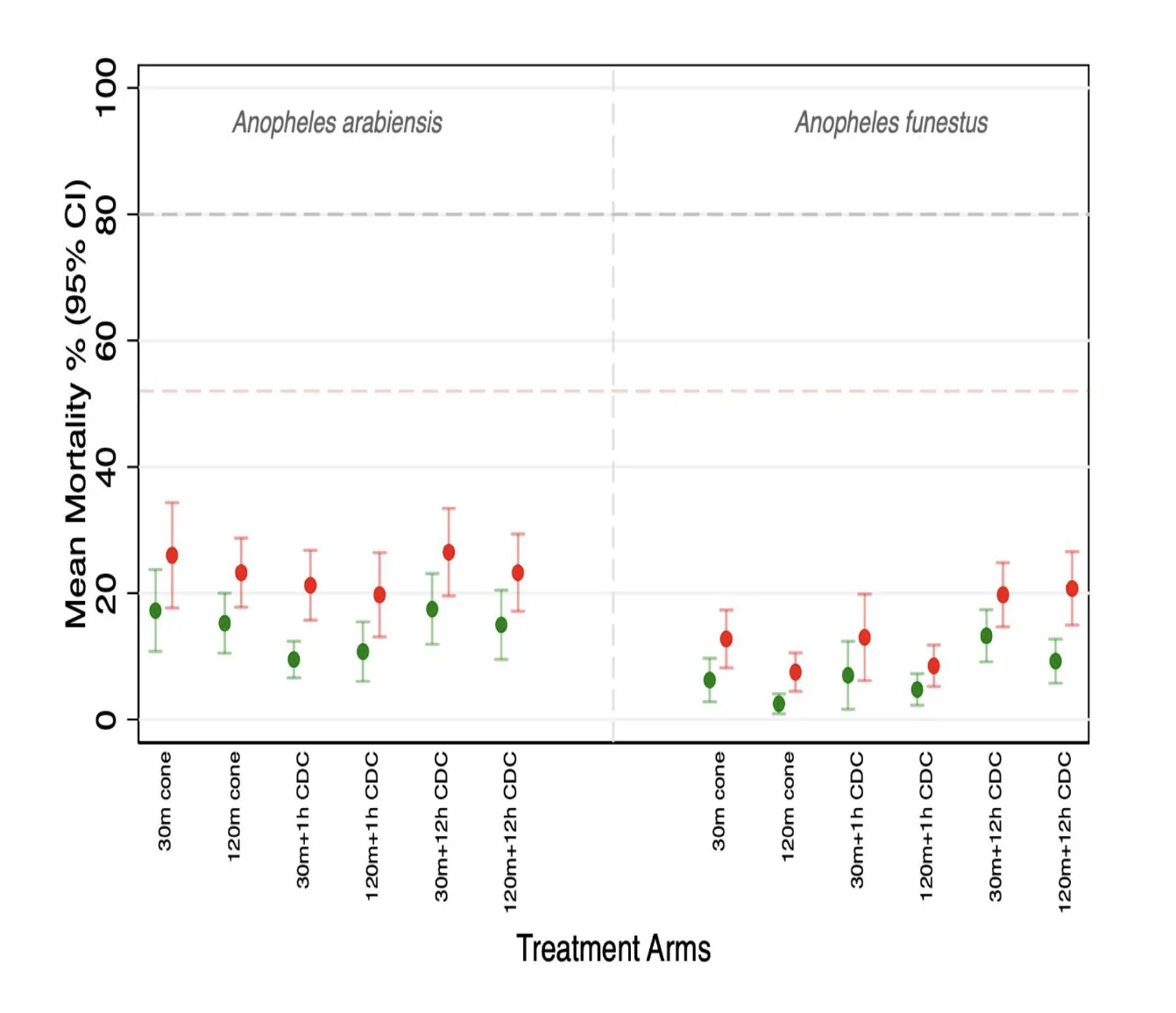
Delayed mosquito mortality at 72 hrs (green) and 120 hrs (red) in the flight cone bioassay for pyrethroid-resistant *An. arabiensis* and *An. funestus*. The black dotted line indicates the WHO threshold level (80%) and the red dotted line represents the indicative value (52%).

Species differences were observed, where *An. arabiensis* consistently showed higher mortality than *An. funestus* at both 72 hrs (9.5-17.5% vs 2.5-13.2%, respectively) and 120 hrs (19.8-26.5% vs 7.5-20.8%, respectively). Although, both species showed increased delayed mortality, the increase was more marked in *An. funestus* (Figure 5 and Supplementary Table 1).

### Delayed mosquito mortality following cone bioassay with and without the host

At 72 hrs post-exposure, mosquito mortality varied by exposure duration and host presence. Under the reference condition (30-min exposure without rabbit), mortality was 12.6%. The presence of a rabbit during 30-min exposure reduced mortality to 10.1% (OR = 0.77, 95% CI: 0.56–1.06, p =

0.107). Extending exposure to 12 hrs without a rabbit increased mortality to 15.4%, although this was not statistically significant (OR = 1.28, 95% CI: 0.95–1.71, p = 0.103). In contrast, 12-hrs exposure with a rabbit significantly increased mortality to 23.5%, more than doubling the odds of death compared with the reference (OR = 2.25, 95% CI: 1.71–2.97, p < 0.001) (Table 3).

At 120 hrs post-exposure, mortality increased across all treatments, ranging from 22.6% to 31.9%. Compared with the reference condition (30-min exposure without rabbit; 22.6%), 30-min exposure with a rabbit resulted in a modest, non-significant increase in mortality (24.0%; OR = 1.09, 95% CI: 0.85–1.39, p = 0.494). Extending exposure to 12 hrs without a rabbit increased mortality to 27.4%, with a significant increase in the odds of death (OR = 1.33, 95% CI: 1.04–1.68, p = 0.021). The highest mortality was observed after 12-hrs exposure in the presence of a rabbit (31.9%), with rabbit presence significantly increasing the odds of death (OR = 1.69, 95% CI: 1.34–2.14, *p* < 0.001) (Table 3).

**Figure 6:**
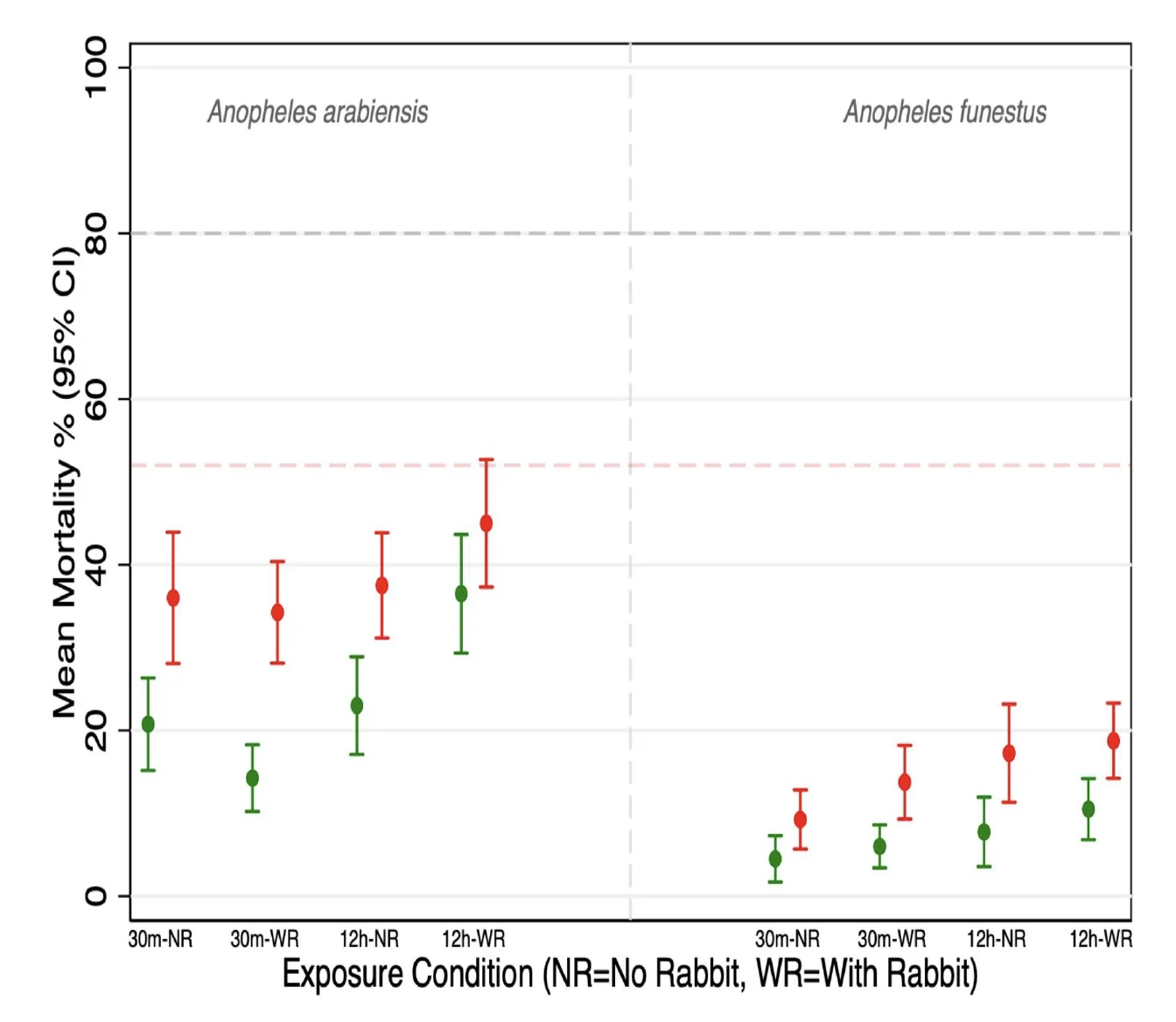
Delayed mosquito mortality at 72 hrs (green) and 120 hrs (red) in the cone bioassay with and without host (rabbit) for pyrethroid-resistant *An. arabiensis* and *An. funestus*. The black dotted line indicates the WHO threshold level (80%) and the red dotted line represents the indicative value (52%).

Species differences were again observed, with a consistently higher mortality in *An. arabiensis* than *An. funestus* at both 72 hrs (14.2–36.5% vs 4.5–10.5%, respectively) and 120 hrs (34.2–45.0% vs 9.2–18.8%, respectively). However, at 120 hrs, *An. funestus* showed consistent and increased mortality across all treatment conditions (Figure 6 and Supplementary Table 2).

### Meta-analysis

The indicative value estimated from the meta-analysis was 52% (95% Confidence Interval: 41, 66%). Overall, mortality remained below the ±5% indicative value across all treatment conditions, suggesting that none of the treatment conditions reliably assessed the bioefficacy of chlorfenapyr-IRS. Observed mosquito mortality in the cone bioassay, both with and without rabbit, ranged from 12.6 to 23.5% at 72 hrs and 22.6 to 31.9% at 120 hrs.

## Discussion

Indoor residual spraying with chlorfenapyr (Sy-lando® 240SC) is an important tool for malaria vector control, particularly in areas with high pyrethroid resistance where chlorfenapyr ITNs are not in use [18,35]. However, assessing the bioefficacy of chlorfenapyr on treated walls using the standard 30-min cone bioassay remains challenging, as cone bioassays consistently underestimate chlorfenapyr efficacy [14,18,23]. This study investigated a modified cone bioassay to better capture the bioefficacy of Sylando® 240SC applied to mud surface, by assessing the effect of extended cone exposure time, post-exposure mosquito confinement in a clean CDC bottle, and host presence (rabbit) in a test room. To capture the full effect of chlorfenapyr, delayed mortality was monitored from 72 to 120 hrs. While some studies extend observations to 168 hrs [19], assessments in this study ended at 120 hrs. Although the results did not differ substantially from a traditional cone bioassay, suggesting this specific method may not be recommended for operational monitoring, the design provides a mechanistic insight into how host-seeking behaviour and mosquito activity influence chlorfenapyr performance.

The relatively low mortality observed in this study is consistent with the known mode of action of chlorfenapyr. Unlike neurotoxic insecticides that cause rapid knockdown, chlorfenapyr acts as a pro-insecticide that disrupts mitochondrial respiration after metabolic activation [16]. As a result, toxicity is strongly influenced by mosquito metabolic activity and typically results in delayed mortality [23]. Because the standard cone bioassay restricts mosquito movement and limits flight [14,18,19,23,36], it may not provide sufficient conditions for full activation of chlorfenapyr, which may have contributed to the modest mortality observed in this study.

The addition of a clean CDC bottle bioassay was intended to stimulate mosquito activity after contact with the treated surface, as it is not easy for the mosquitoes to rest on its smooth surface and we anticipated that it would promote movement and allow time for metabolic activation [24]. However, the CDC bottle did not consistently improve mosquito mortality. This may be because mosquitoes, after being transferred from the cone to the CDC bottles, were quite still after an initial period of flight and rested most of the time at the bottom of the CDC bottles, as is often observed in untreated control CDC bottle bioassays when doing susceptibility tests, resulting in insufficient sustained activity to increase metabolic rate and promote chlorfenapyr bioactivation. The findings therefore do not support the addition of a post-cone holding period in clean CDC bottles to increase the conversion of chlorfenapyr to its metabolite.

While 12 hrs of exposure with a rabbit significantly increased mosquito mortality, this may reflect a behavioural response to physical or odour cues. However, mosquito mortality still failed to fall within 5% of the indicative value when a rabbit was present, likely reflecting the dominant role of host preference and behaviour. Although *An. funestus* is predominantly anthropophilic and *An. arabiensis* is more zoophilic [37,38], both laboratory strains readily feed on rabbits, suggesting host choice was not a limiting factor. Rather, it is possible that the presence of a small stationary host was insufficient to stimulate mosquito flight activity within the cone. Moreover, the cone bioassay enforces forced contact with the surface and restricts free-flying host-seeking behaviour [14], mosquitoes may quickly rest further limiting insecticide conversion. Although increasing the number of rabbits could theoretically strengthen odour and heat plumes and enhance activation, host attractiveness rather than host quantity alone drives mosquito stimulation. Humans provide stronger and more continuous cues to which *An. funestus* and *An. arabiensis* are highly responsive [39,40]. Therefore, in operational village settings, human presence and movement would likely increase mosquito interaction with chlorfenapyr-treated walls more than a single rabbit in a test room, potentially resulting in higher mortality. Further work using cones applied overnight in experimental huts or local homes with humans moving around inside to explore this hypothesis is warranted.

Cone exposure time showed context-dependent effects across the two experiments, indicating that duration alone was insufficient to consistently increase chlorfenapyr's efficacy without concurrent mosquito activity [14,18,23,36]. In the flight cone bioassay, increasing exposure time from 30 to 120 minutes did not improve and occasionally reduced mosquito mortality at both 72 and 120 hrs. This suggests that prolonged static contact with chlorfenapyr-treated mud surface limits mosquito activity, thereby reducing the metabolic conversion of chlorfenapyr into its lethal metabolite [14,18,23]. Instead, mortality was modestly increased with extended post-exposure confinement in a clean CDC bottle, indicating that the bottle environment partially stimulated mosquito movement. In contrast, extending exposure time from 30 minutes to 12 hours in the host-cone bioassay consistently increased mortality, especially in the presence of a host, where behavioural stimulation occasionally activated the mosquitoes, increasing movement and enhancing metabolic activation.

Overall, mortality at 72 hours was consistently higher in *An. arabiensis* than in *An. funestus*, reflecting the differences in resistance level between the species (Table 1). This difference was also in line with findings from a recent study conducted in the same facility. On mud substrates, chlorfenapyr induced 66.2% mortality in *An. arabiensis* and 39.5% in *An. funestus* at 72 hrs over 12 months using a miniature experimental hut (MEH) with a rabbit host [19]. The comparatively higher mortality observed likely reflects differences in assay design, as the MEH permits free flight and host-seeking behaviour, thereby enhancing metabolic activation, whereas the cone bioassay used in this study restricts mosquito movement. Notably, both studies show a consistent increase in mortality over time for both species, indicating the slow-acting nature of chlorfenapyr and its conversion to lethal metabolite is observed over longer duration holding time [23].

### Limitations

This study had two limitations. First, because chemical deposition on the filter papers was not verified, the actual chlorfenapyr dose may have been lower than anticipated. Second, mosquitoes surviving or succumbing to exposure were not collected in Eppendorf microcentrifuge tubes for downstream biochemical analysis. Chlorfenapyr is a pro-insecticide that requires metabolic bioactivation by cytochrome P450 monooxygenases into its toxic form, tralopyril. Because we did not quantify the relative recovery of parent chlorfenapyr versus bioactivated tralopyril within individual insects, we cannot definitively conclude whether instances of low mortality were caused by insufficient metabolic conversion or by phenotypic recalcitrance to the treated substrates. Future evaluations would benefit from separate molecular profiling of dead and surviving cohorts to isolate these biochemical mechanisms.

An important consideration extending beyond this study is the increasing deployment of chlorfe-napyr-based dual-active ingredient ITNs across sub-Saharan Africa [41]. These dual AI nets have demonstrated superior efficacy over standard pyrethroid and pyrethroid-PBO ITNs in reducing malaria incidence [42,43], making them a cornerstone of current vector control strategies. However, co-deploying chlorfenapyr-based IRS in the same operational environments could significantly increase selection pressure, potentially accelerating the development of malaria mosquito resistance and compromising the long-term effectiveness of this vital chemical class. To preserve chlorfenapyr susceptibility, its use in IRS programs should be carefully balanced within an integrated insecticide resistance management (IRM) framework [44]. This might include strategically restricting chlorfenapyr-based IRS to targeted epidemiological settings, such as internally displaced persons (IDP) camps, or prioritising alternative chemical classes for standard IRS campaigns where next-generation ITNs are already widely distributed.

## Conclusions

This study demonstrates small and context-dependent changes in delayed mortality associated with prolonged exposure and increased mosquito activity, consistent with the role of metabolic activation in chlorfenapyr efficacy. However, these effects were limited and inconsistent and did not result in mortality levels indicative of improved bioefficacy or support the use of these modifications for operational monitoring. Further work is required to evaluate whether more behaviourally realistic exposure conditions can improve the measurements of chlorfenapyr IRS performance.

## Appendix

**Supplementary Table 1:** Delayed mortality at 72 and 120 hours of *An. funestus* and *An. arabiensis* following cone exposure on chlorfenapyr-treated mud surfaces and CDC bottle confinement.

Mosquito species (holding time)	Treatment arms		Total exposed per test day	No. dead	% Arithmetic mean mortality (95% CI)	OR* (95% CI)	p-value
	Cone-exposure time (mins)	Post-exposure activity time (hrs) in a clean CDC bottle					
*An. arabiensis* (72 hrs)	30	None	400	69	17.2 (11.0 – 23.5)	1.00	
	120	None	400	61	15.2 (10.6 – 19.9)	0.86 (0.59 – 1.26)	0.453
	30	1 hr CDC	400	38	9.5 (6.7 – 12.3)	0.49 (0.32 – 0.76)	0.001
	120	1 hr CDC	400	43	10.8 (6.2 – 15.3)	0.57 (0.37 – 0.86)	0.008
	30	12 hrs CDC	400	70	17.5 (12.1 – 22.9)	1.02 (0.70 – 1.48)	0.924
	120	12 hrs CDC	400	60	15.0 (9.7 – 20.3)	0.84 (0.57 – 1.24)	0.379
*An. funestus* (72 hrs)	30	None	400	25	6.2 (2.9 – 9.6)	1.00	
	120	None	400	10	2.5 (1.0 – 4.0)	0.38 (0.18 – 0.81)	0.012
	30	1 hr CDC	400	28	7.0 (1.8 – 12.2)	1.13 (0.64 – 1.99)	0.667
	120	1 hr CDC	400	19	4.8 (2.3 – 7.2)	0.74 (0.40 – 1.38)	0.350
	30	12 hrs CDC	400	53	13.2 (9.2 17.3)	2.34 (1.41 – 3.87)	0.001
	120	12 hrs CDC	400	37	9.2 (5.8 – 12.7)	1.54 (0.91 – 2.63)	0.111
*An. arabiensis* (120 hrs)	30	None	400	104	26.0 (17.9 – 34.1)	1.00	
	120	None	400	93	23.2 (17.9 – 28.6)	0.85 (0.61 – 1.19)	0.350
	30	1 hr CDC	400	85	21.2 (15.9 – 26.6)	0.75 (0.54 – 1.06)	0.102
	120	1 hr CDC	400	79	19.8 (13.3 – 26.2)	0.68 (0.48 – 0.96)	0.030
	30	12 hrs CDC	400	106	26.5 (19.8 – 33.2)	1.03 (0.74 – 1.43)	0.868
	120	12 CDC	400	93	23.2 (17.3 – 29.2)	0.85 (0.61 – 1.19)	0.350
*An. funestus* (120 hrs)	30	None	400	51	12.8 (8.3 – 17.2)	1.00	
	120	None	400	30	7.5 (4.5 – 10.5)	0.54 (0.34 – 0.88)	0.013
	30	1 hr CDC	400	52	13.0 (6.4 – 19.6)	1.02 (0.67 – 1.56)	0.914
	120	1 hr CDC	400	34	8.5 (5.3 – 11.7)	0.63 (0.39 – 1.00)	0.049
	30	12 hrs CDC	400	79	19.8 (14.8 – 24.7)	1.73 (1.17 – 2.56)	0.006
	120	12 hrs CDC	400	83	20.8 (15.1 – 26.4)	1.85 (1.25 – 2.73)	0.002

* Adjusted Odds Ratio

**Supplementary Table 2:** Delayed mortality at 72 and 120 hours of *An. funestus* and *An. arabiensis* following cone exposure on chlorfenapyr-treated mud surfaces and CDC bottle confinement.

Mosquito species (holding time)	Treatment arms	Total exposed per test day	No. Dead	% Arithmetic mean mortality (95% CI)	OR*(95% CI)	*p*-value
*An. arabiensis* (72 hrs)	Treated panel 30 min no rabbit	400	83	20.8 (15.3 – 26.2)	1.00	
	Treated panel 30 min with rabbit	400	57	14.2 (10.3 – 18.2)	0.63 (0.43 – 0.91)	0.015
	Treated panel 12 hrs no rabbit	400	92	23.0 (17.3 – 28.7)	1.15 (0.81 – 1.61)	0.433
	Treated panel 12 hrs with rabbit	400	146	36.5 (29.5 – 43.5)	2.28 (1.65 – 3.15)	<0.001
*An. funestus* (72 hrs)	Treated panel 30 min no rabbit	400	18	4.5 (1.8 – 7.2)	1.00	
	Treated panel 30 min with rabbit	400	24	6.0 (3.5 – 8.5)	1.36 (0.72 – 2.55)	0.342
	Treated panel 12 hrs no rabbit	400	31	7.8 (3.7 – 11.8)	1.79 (0.98 – 3.26)	0.057
	Treated panel 12 hrs with rabbit	400	42	10.5 (6.9 – 14.1)	2.51 (1.41 – 4.44)	0.002
*An. arabiensis* (120 hrs)	Treated panel 30 min no rabbit	400	144	36.0 (28.3 – 43.7)	1.00	
	Treated panel 30 min with rabbit	400	137	34.2 (28.3 – 40.2)	0.92 (0.68 – 1.24)	0.589
	Treated panel 12 hrs no rabbit	400	150	37.5 (31.3 – 43.7)	1.07 (0.79 – 1.45)	0.646
	Treated panel 12 hrs with rabbit	400	180	45.0 (37.5 – 52.5)	1.51 (1.12 – 2.03)	0.007
*An. funestus* (120 hrs)	Treated panel 30 min no rabbit	400	37	9.2 (5.8 – 12.7)	1.00	
	Treated panel 30min with rabbit	400	55	13.8 (9.4 – 18.1)	1.58 (1.01 – 2.46)	0.045
	Treated panel 12hrs no rabbit	400	69	17.2 (11.5 – 23.0)	2.07 (1.35 – 3.19)	0.001
	Treated panel 12hrs with rabbit	400	75	18.8 (14.3 – 23.2)	2.30 (0.50 – 3.52)	<0.001

* Adjusted Odds Ratio

**Supplementary Table 3:** Meta-analysis table.

Database	Search keywords	Studies identified	Eligible studies included	Inclusion criteria
PubMed	("chlorfenapyr") OR ("Sylando 240SC") OR ("pyrrole") AND ("indoor residual spraying") AND ("pyrethroid-resistant mosquito")	7 records	2 studies	1: Community trials (wall cone bioassay); 2: Experimental hut trials (wall cone bioassay); 3: Ifakara Ambient Chamber Test (I-ACT); 4: Resistant *Anopheles* mosquito species; 5: Surface sprayed with chlorfenapyr.
Google scholar	("chlorfenapyr" OR "Sylando 240SC" OR "pyrrole" AND "indoor residual spraying" AND pyrethroid-resistant mosquito)	612 records	3 studies	
